# Exploring factors that influence the knowledge and awareness of breastfeeding among Saudi mothers: a qualitative study

**DOI:** 10.3389/fnut.2025.1516686

**Published:** 2025-03-05

**Authors:** Amany Anwar Saeed Alabdullah, Nasreen Mohammed ALshamy, Layan Majed Alzahrani, Rana Abdullah Safhi, Mzoun Turki Alrashed, Layan Munahi Al-Mukhtalah, Marwa Anwar Alenazi, Rana Saeed Alzahrani, Ahad Turki Alshammari, Fayzah Hussain Alhussain, Manal Awn Alharthi, Fidaa Mohammed Alsaran

**Affiliations:** ^1^Department of Maternity and Pediatric Nursing, College of Nursing, Princess Nourah Bint Abdulrahman University, Riyadh, Saudi Arabia; ^2^Maternal and Child Health Care Department, College of Nursing, King Saud University, Riyadh, Saudi Arabia; ^3^College of Nursing, Princess Nourah Bint Abdulrahman University, Riyadh, Saudi Arabia

**Keywords:** knowledge, awareness, breastfeeding, mothers, Saudi Arabia

## Abstract

**Purpose:**

Breastfeeding provides short- and long-term benefits for mothers and babies. Despite these advantages, the prevalence of breastfeeding among Saudi mothers is low. We explored factors affecting the knowledge and awareness of breastfeeding among Saudi mothers in Riyadh.

**Methods:**

This qualitative study included 17 mothers. The inclusion criteria were Saudi mothers aged 18–50 years, with at least one child, living in Riyadh. Semi-structured interviews of approximately 20–40 min were conducted online or in person between March and April 2024. Data were analyzed using the constant comparative method with NVivo 11. Ethical approval was obtained from an academic institutional review board prior to data collection.

**Results:**

Two key themes emerged from the data. Theme 1 concerned factors facilitating knowledge and awareness of breastfeeding; subthemes included positive experience with breastfeeding, knowledge of breastfeeding, family support, and financial considerations. Theme 2 concerned factors limiting knowledge and awareness of breastfeeding; subthemes included negative experiences of breastfeeding, work situations, misconceptions, and mental health issues.

**Conclusion:**

Despite the low prevalence of breastfeeding in Saudi Arabia, mothers were aware of its benefits. Physicians and midwives should provide ongoing education and support for mothers during ante- and post-natal periods to minimize misconceptions regarding breastfeeding and promote its use.

## Introduction

Breastfeeding confers considerable health benefits for infants and mothers (1). According to Bermejo-Haro et al. (2), breastfeeding provides essential nutrients, antibodies, and hormones necessary for infants’ growth and the development of their immune systems. Other benefits for infants include minimizing the risk of infection and allergies and enhancing their cognitive development (3, 4). For mothers, breastfeeding is crucial for reducing the risk of breast and ovarian cancers, as well as enhancing postpartum recovery and helping to lose post-pregnancy weight (5). In addition, breastfeeding plays a vital role in fostering a maternal–child bond (Joseph, 2020). Although breastfeeding has these positive attributes, the prevalence of breastfeeding in Saudi Arabia remains low. According to Alyousefi (6), just 28% of mothers practiced exclusive breastfeeding for the first six months. This compares with the global prevalence of 48% in 2023 (7).

Within Saudi Arabia, breastfeeding practices are closely associated with cultural, economic, and social dimensions (6, 8). From a historical perspective, the culture of Saudi Arabia emphasizes the need for breastfeeding (9). Traditional practices and religious teachings also emphasize the importance of breastfeeding until the age of two years (10). However, Saudi Arabia has recently experienced a change in the dynamics associated with breastfeeding (11). Prelacteal feeding, which includes the introduction of formula milk and the delayed initiation of breastfeeding, has had a considerable impact on mothers and influenced their decision not to breastfeed their children (12, 13).

The World Health Organization (WHO) has emphasized the need for breastfeeding for at least the first six months of an infant’s life; however, this trend has not been followed within Saudi Arabia in recent years (9). Several factors are crucial to understanding the low breastfeeding rate in Saudi Arabia. These include unhelpful hospital policies, such as not enforcing policies that encourage breastfeeding; healthcare staff who need additional training to effectively support women to breastfeed; the lack of ongoing social support; and the tensions associated with breastfeeding, for example when mothers have to juggle multiple roles, for example combining looking after their baby with a return to work or study (14, 15). A better understanding of these factors would assist in the development of interventions and policies to increase breastfeeding rates. Although some research has investigated breastfeeding in Saudi Arabia, many factors still need to be addressed (6). Against this backdrop, our current study aimed to address these gaps in our knowledge, in particular with regard to factors affecting breastfeeding knowledge and awareness in Saudi Arabia. This study examined facilitators of and barriers to breastfeeding in Riyadh, Saudi Arabia.

## Materials and methods

We used a qualitative design to comprehensively explore which factors influenced the knowledge and awareness of breastfeeding among Saudi mothers. This approach provided rich and detailed data on the phenomena and allowed us to better understand current breastfeeding trends (16). We approached Saudi mothers in two ways: outreach in malls and in mosques. These avenues were chosen to ensure a broad representation of mothers living in Riyadh, who were accessible through these community-based networks. The outreach included providing detailed information about the study, such as its goals, eligibility criteria, and instructions on how to participate. Out of 83 mothers initially approached, 52 expressed a willingness to participate. The researchers then began conducting interviews until theoretical saturation was reached. Saturation occurred after 17 transcripts had been analyzed, as no new major themes or information emerged, suggesting that the data was sufficiently rich and diverse for the study’s objectives. Thus, the final sample size was 17 Saudi mothers. Although the sample size was small, it allowed for a detailed exploration of the participants’ views and opinions, as it reached theoretical saturation— the point at which gathering more data *“reveals no new properties nor yields any further theoretical insights*” (17, p. 345). The study inclusion criteria were any Saudi mothers aged between 18 and 50 years who had at least one child and lived in Riyadh. Any participant who did not meet these criteria was excluded. A purposive sampling technique was used. Two experts in research and midwifery reviewed the research instrument and formulated the following interview questions based on a pilot study:

- What is your source of knowledge and awareness about breastfeeding?
- How did this source contribute to your experience of breastfeeding?
- What factors affected your choice of how to feed your baby?
- What other factors affected your knowledge and experience regarding breastfeeding?

Data were collected through semi-structured, audio-recorded interviews that were conducted privately, online or in person, between April and May 2024. The interviews continued until the collected data were repeated and theoretical saturation was reached. Prior to the interviews, the participants signed informed consent forms. Each interview took between 20 and 40 min, and the participants were asked for their opinions about breastfeeding.

The recorded data were transcribed and analyzed using NVivo 11 software. We used the constant comparative method, applied iteratively as new data were collected. This approach allows researchers to compare transcripts for emerging themes and subthemes as they closely examine the data from each transcript and among transcripts to identify common themes, subthemes, ideas, and meanings that emerge repeatedly and form patterns (18).

Ethical approval for this study was obtained from an academic institution in March 2024. Participants were informed about the details of the study, of their right to withdraw from it at any time and for any reason, and of the recording and anonymous coding of the interviews, after which their written consent to participate in this study and for the recording of the interviews was obtained. Data confidentiality and privacy were maintained at all times.

## Results

The factors we investigated were the facilitators of knowledge and awareness of breastfeeding and the barriers to knowledge and awareness of breastfeeding. Table 1 shows the themes and subthemes that emerged from the interviews.

**Table 1:** Themes and subthemes of breastfeeding that emerged from the interviews.

Theme	Subtheme
Facilitators of knowledge and awareness of breastfeeding	Positive experience with breastfeeding Knowledge of breastfeeding Family support Financial considerations
Barriers to knowledge and awareness of breastfeeding	Negative experience of breastfeeding Working situation Misconceptions Mental health issues

### Theme 1: facilitators of knowledge and awareness of breastfeeding

This theme comprised four subthemes: positive experience with breastfeeding, knowledge of breastfeeding, family support, and financial considerations.

#### Subtheme 1: positive experience with breastfeeding

Fifteen participants emphasized this subtheme as the main factor in their decision to breastfeed and to continue breastfeeding. They considered breastfeeding enhanced their children’s health, their health, and their mother–child bonding. Participant 9 (P9) spoke of the “positive result I observed for me and my baby”.

P16 agreed that breastfeeding had benefited her first child’s health: “My decision was due to the results of breastfeeding my child, which provided him with vitamins and immune strength, and the positive effects of increased bonding and emotion between me and my child.” Moreover, P1 attributed her decision to continue breastfeeding to the “feeling of connection between me and the child. I loved that feeling”.

P14 compared the difference between her previous baby, whom she had formula-fed, and her 10-month-old baby, whom she was breastfeeding exclusively. She “noticed a very big difference between them in terms of perceptions and attitudes to surroundings, overall well-being, and health.” P3 and P17, who each had one child, agreed that breastfeeding had a positive effect on the mother in terms of rapid postpartum weight loss and an improved “psychological state” (P17). P7 suggested breastfeeding was “the best gift you can give to your child”.

#### Subtheme 2: knowledge of breastfeeding

In this subtheme, P2, P3, P4, P5, P6, P7, P8, and P17, who were of different ages and levels of education and experience, highlighted the importance of acquiring knowledge about correct breastfeeding positions and latching on. For example, P3 noted, “This knowledge contributed to strengthening my confidence in breastfeeding” (P3). Family members, as well as members of the community and healthcare professionals, were critical sources of knowledge. P17 stated, “My doctor provided practical consultations on the correct position for me and my child during breastfeeding and explained to me […] the benefit of breastfeeding my child.” P4 and P7 received knowledge about breastfeeding during their antenatal care and immediately after childbirth. According to P4, this “influenced my decision” (P4) to breastfeed her most recent child.

P2, P3, P4, P5, P6, P7, and P8 said that they received knowledge about the importance of breastfeeding and its benefits for mothers and their neonates directly from physicians and midwives. P5 and P8 gained their knowledge from various social media platforms. Midwives and physicians use these platforms to teach breastfeeding techniques and the benefits of breastfeeding, and P5 and P8 felt accessing these platforms was convenient. P5 emphasized the importance of easily accessible online resources in “educating and empowering me” and helping to promote and facilitate breastfeeding practices.

The participants’ responses demonstrated that gaining knowledge in person or via social media from healthcare professionals’ statements about breastfeeding played a crucial role in determining what mothers thought about matters such as “reducing my weight” and “improving child immunity” (P6). Participants found this knowledge useful and offered other reasons for supporting breastfeeding, including “minimizing the risk of breast cancer” (P3).

The knowledge they acquired led some Saudi mothers to adopt and continue with successful breastfeeding practices; as P3 stated, these were “positive reasons that encouraged me to breastfeed”.

#### Subtheme 3: family support

In this subtheme, two participants highlighted the major influence and positive impact of family support on breastfeeding decisions. P6, who had five children, received considerable support, especially with her first child (the first grandchild in the family). P17, who had only one child, mentioned that her “sister was her backbone during the beginning of the breastfeeding journey.”

#### Subtheme 4: financial considerations

Financial income can impact the decision to breastfeed because breastfeeding is the cheapest method of feeding. However, only one participant (P10), who had just finished her bachelor’s degree and had one child, mentioned being able to save more money “to take care of myself and my child” as a factor in her decision to breastfeed.

### Theme 2: barriers to knowledge and awareness of breastfeeding

Theme 2 comprised four subthemes: negative breastfeeding experiences, working situations, misconceptions, and mental health issues.

#### Subtheme 1: negative experience of breastfeeding

Eight participants (P1, P2, P4, P5, P7, P8, P12, and P17) mentioned insufficient education and awareness of breastfeeding, especially during their early pregnancies (i.e., with their older children). P8 spoke of “a lack of awareness of the health benefits and how to breastfeed my first two babies.” P1 insisted that education should be ongoing, starting before pregnancy and continuing until after birth. She stated that for exposure to breastfeeding education, “one time is not enough.”

P12 emphasized her negative experience with breastfeeding, raising the issue of starting breastfeeding late due to insufficient awareness and knowledge: “Unfortunately, my baby rejected breastfeeding because, at birth, I started bottle feeding. Nobody encouraged me to breastfeed, so the infant became accustomed to it and relied on it, and it was difficult to breastfeed later.”

#### Subtheme 2: working situation

P2, P3, P15, and P17 mentioned that work was one of the factors influencing their failure to continue breastfeeding exclusively. P17 said that “returning to work two months after giving birth made me consider formula feeding along with breastfeeding, especially as there is no time or place to pump my breast milk,” whereas P3 stated that “living in crowded Riyadh makes it difficult to breastfeed. It took me 2 h [of] transportation to leave home and get back to my baby, and I came home very exhausted from the traffic and workload.”

#### Subtheme 3: misconceptions

Three participants, P1, P5, and P15, stated that various misconceptions they had heard from their mothers or other family members affected their decisions to breastfeed exclusively or also to use formula. P1 shared her personal experience of her first pregnancy, highlighting the impact of misconceptions acquired from her mother, namely, “do not conceal the child’s face while breastfeeding” and “do not hold your breast while breastfeeding.” In addition, P15 shared that she thought her “breast shape would change and look very ugly.”

#### Subtheme 4: mental health issues

Mental health was one of the factors that affected a mother’s decision to introduce formula feeding. Two participants mentioned how postpartum depression influenced their decision not to breastfeed and introduce formula milk. P1 highlighted the psychological changes that mothers undergo, which can contribute to feelings of “depression, fear, and mixed emotions related to motherhood and the responsibilities it entails.” Another participant (P15) said, “I just got married and became pregnant. I could not accept all the changes in my body, and I felt I could not care for my child or breastfeed, it was too much.”

## Discussion

This study investigated various factors that acted as facilitators and barriers to breastfeeding in Saudi Arabia. Here, we also compare our findings with relevant reports from the literature, although we did not conduct a full literature review. In defining the facilitators of breastfeeding, researchers refer to factors that encourage breastfeeding among mothers (19). In contrast, barriers to breastfeeding are factors that hinder and discourage mothers from breastfeeding their infants (20). Personal breastfeeding experiences can impact a mother’s decision to continue or discontinue breastfeeding (21). If these experiences were positive, then the result is a strong commitment to breastfeeding, but if they were negative, this could have a corresponding negative impact on their attitude toward breastfeeding (22). In our study, the participants outlined a sense of positivity when it came to shaping their attitudes toward breastfeeding through their acquisition of positive firsthand experiences. As shown by our findings, mothers who experienced considerable health benefits and strengthened mother–child bonds through breastfeeding were likely to continue breastfeeding their children. These findings align with those of Alyousefi (6), who discussed the benefits of breastfeeding and how it can promote maternal and infant health. Personal experiences are, therefore, crucial in validating a mother’s practices and serve as powerful motivators for them to continue breastfeeding their children.

The participants in our study highlighted that breastfeeding was crucial for enhancing immunity and reducing the risk of infections in their infants. Our results also indicated that mothers felt breastfeeding was important to them in terms of a more rapid return to their pre-pregnancy weight and a reduced risk of certain cancers. Respondents further stated that breastfeeding was essential for strengthening the bond between mothers and children, particularly by creating a deeper emotional connection. These findings are supported by the results of Bailey (23), who emphasized the importance of skin-to-skin contact during breastfeeding in creating appropriate emotional and psychological development. Overall, the participants in our study reported positive firsthand experiences regarding breastfeeding, including feeling a sense of accomplishment and satisfaction.

Some mothers may require additional knowledge regarding breastfeeding and its associated benefits (24). Knowledge of breastfeeding is generally promoted through education provided by healthcare professionals who can assist in shaping the confidence of mothers and their abilities to breastfeed successfully (25). Our findings highlighted the role of mothers’ knowledge of breastfeeding and how it influenced their breastfeeding decisions. As indicated by the respondents, education about correct breastfeeding techniques, including current advice regarding positions and effective latching, was vital for boosting their confidence in breastfeeding and increasing the likelihood of successful breastfeeding. Obtaining accurate information involves acquiring knowledge from healthcare providers about correct breastfeeding techniques, which subsequently promotes breastfeeding ability. In addition, education about correct breastfeeding latch techniques is important when it comes to effective milk transfer, as well as other issues, such as nipple pain and an inadequate supply of milk. Proper latching is vital to ensure the baby receives adequate nutrition; it also reduces the risk of complications, thus reinforcing the mother’s commitment to breastfeeding. Our study highlighted the importance of education targeted at effectively enhancing breastfeeding. Such education programs should involve hands-on demonstrations, as well as the use of visual aids and useful tips. Our findings also showed the importance of education during the antenatal period, with continuous follow-up and support after delivery and beyond, to enhance successful breastfeeding among mothers.

Another important factor is the support that breastfeeding mothers acquire from their families (25). If the support is appropriate, there will be positive reinforcement toward breastfeeding, encouraging its continuation. Our study’s findings highlighted the vital role of the family in supporting breastfeeding among mothers in Saudi Arabia. Encouragement, support, and assistance from family members are important elements in creating a positive, fostering environment for breastfeeding. These findings align with those reported by Aledreesi and Omar (26), who indicated that family support could have a significant positive influence on breastfeeding.

Family support exists in various forms, including emotional reassurance, practical assistance, and positive reinforcement. Practical assistance the family provides to the mother is also crucial, including assisting mothers with household tasks, offering to care for children, and providing helpful physical support during breastfeeding. This form of assistance plays a key role in enhancing a mother’s focus on breastfeeding and simultaneously reducing the stress of managing multiple responsibilities. Our findings indicated that family members, specifically sisters and mothers, actively supported breastfeeding, sharing responsibilities with and encouraging mothers to breastfeed. These findings agree with those of Theodorah and Mc’Deline (27), who found that family members provided invaluable support for mothers, helping them to continue breastfeeding exclusively. This type of support is essential for creating a nurturing atmosphere that reinforces a mother’s decisions regarding breastfeeding.

Another factor to consider is finance because breastfeeding is considered a cost-effective method compared with formula feeding. This is especially the case for lower-income households for whom cost savings are crucial (28–30). Our results indicated that financial factors are critical in shaping Saudi mothers’ views on breastfeeding. A participant in our study indicated the economic benefits of breastfeeding, with respondents arguing that breastfeeding was more cost-effective than formula feeding. This is because of the cost of purchasing formula, bottles, and other feeding supplies. As indicated by Murad (31), these financial benefits are particularly important for families on a tight budget, for whom the reduced cost associated with breastfeeding can result in greater financial stability. Economic pressures can, therefore, influence breastfeeding, especially for families with limited financial resources who must manage their household expenses effectively.

However, certain factors act as barriers to mothers’ breastfeeding, including negative experiences of breastfeeding, which can result in mothers discontinuing breastfeeding (32, 33). Our findings showed that negative experiences related to breastfeeding included a lack of timely education and support, which can influence the duration of breastfeeding. The perceptions of individuals with adverse breastfeeding outcomes could be attributed to a lack of timely education. This included insufficient knowledge regarding the initiation of early breastfeeding following delivery, as well as a lack of knowledge about appropriate breastfeeding techniques, latching on, and positioning, all of which can result in difficulties when encouraging new mothers to breastfeed.

These results align with those reported by Aledreesi and Omar (26), who indicated that inadequate education can lead to a variety of issues, such as nipple pain and poor milk production, which can result in the early cessation of breastfeeding. In addition, physical discomfort, inadequate support, and perceived feeding problems result in many individuals discontinuing breastfeeding. Thus, there is a need for timely education and support to help mothers deal with the negative experiences that may be associated with breastfeeding. Education during the prenatal phase, such as providing basic practical guidance and overcoming common challenges, is crucial in preparing mothers for the realities of breastfeeding (34–36). The healthcare system should implement appropriate practices, specifically by employing well-trained midwives who can enhance prenatal education, as well as provide immediate postnatal support, accessible lactation consultations, and education for family members who may be involved in breastfeeding (37).

A mother’s work situation is another factor that can pose a challenge to exclusive breastfeeding. Our study revealed the importance of balancing work responsibilities and breastfeeding. In addition, the work environment can impact most working mothers’ abilities to continue breastfeeding exclusively. These challenges show the importance of managing work and breastfeeding and the need to provide supportive workplace policies and flexible work arrangements. These findings align with the existing literature, showing that workplace constraints are the main factors that result in a reduced duration of breastfeeding. For example, a study involving hospital employees in Thailand found that 76% of mothers continued breastfeeding before returning to work, but 24% had to stop breastfeeding within three months of returning to work (38). It is important for breastfeeding mothers that workplaces have supportive policies and flexibility regarding hours, with appropriate facilities for breastfeeding and expressing milk (39, 40).

The participants in our study reported various obstacles to continuing breastfeeding exclusively when they returned to work. The main challenges highlighted included limited time for breastfeeding, a lack of appropriate facilities for expressing milk and breastfeeding, and pressures from the workplace that discouraged mothers from breastfeeding. These findings are consistent with other reports in the literature, including studies of working mothers’ experiences of exclusive breastfeeding in Ghana and Kenya (41, 42). The difficulty of balancing work with breastfeeding is exacerbated by various demands placed on mothers due mainly to the work environment, where long hours and inflexible schedules can hinder the ability of a mother to maintain exclusive breastfeeding (43). To solve some of these issues, it is important to create flexible working hours or remote working arrangements that allow work and breastfeeding to be managed effectively (44). Additionally, there is a need for lactation breaks; ensuring that mothers have sufficient time and privacy to express milk during work hours can support continued breastfeeding (45, 46). This can be achieved by providing comfortable and private breastfeeding and pumping rooms within the workplace. Regarding the workplace, employers and policymakers can play key roles in developing an environment that is supportive when it comes to breastfeeding. This includes creating policies to address the challenges of balancing the time spent working with the time needed for breastfeeding (47).

An additional barrier to breastfeeding is the existence of misconceptions and misinformation regarding breastfeeding, which can negatively impact a mother’s decision to breastfeed (8, 15, 48). In our study, the participants articulated that these misconceptions created anxiety and reluctance to breastfeed, leading to the introduction of formula or the early cessation of breastfeeding. Furthermore, participants stated that misinformation regarding breastfeeding undermined their confidence and willingness to continue breastfeeding, resulting in them needing more motivation to overcome common obstacles to breastfeeding. This misinformation originated from opinionated family members, mainly the older generation, who had outdated information, consistent with findings reported elsewhere. For example, a study in Ghana noted that the older generation had misconceptions about breastfeeding and that information about breastfeeding should be provided to grandmothers in addition to mothers (49). Furthermore, the marketing materials for formula sometimes reinforce misconceptions that common infant behavior, such as crying, is a sign of feeding problems that can be ameliorated using formula (40). To address these misconceptions, comprehensive education programs and public health campaigns aimed at breastfeeding mothers and their family members will be necessary. Our findings highlight the need for support programs that involve the entire family in breastfeeding and encouraging mothers. In addition, it is necessary to integrate family support into healthcare practices, which includes recognizing the role of the family unit in breastfeeding (34).

Another factor that can affect the decision to breastfeed is a mother’s mental health issues. This includes a variety of conditions, including postpartum depression, which can impact a mother’s ability to initiate or maintain breastfeeding (50). The participants in our study highlighted the negative effects that poor mental health had on breastfeeding and breastfeeding practices, including anxiety, depression, and stress. Furthermore, they considered that these mental health issues could result in decreased motivation, a sense of inadequacy, and difficulties when it came to managing the demands of breastfeeding. High levels of anxiety and depression can result in mothers finding it challenging to maintain the emotional and physical stamina needed during breastfeeding. As reported by Al-Nassir (11), various symptoms, including low energy, the feeling of being overwhelmed, and impaired concentration, can hinder the abilities of mothers to establish and sustain breastfeeding. It is important to note that breastfeeding can help prevent depressive symptoms in mothers (50). To address mental health issues, mothers should be screened and assessed, and easy access must be provided to mental health services, supportive counseling and therapy, peer support groups, and breastfeeding support networks. Integrating mental health support into maternal care will improve breastfeeding and maternal health. Therefore, there is a need to create a comprehensive support system for dealing with the psychological and physical aspects of maternal care. This should include developing maternal and infant care services that support better breastfeeding practices and overall well-being.

Overall, our findings suggest that, despite Saudi Arabia’s relatively low breastfeeding rates, Saudi mothers generally have a positive attitude toward breastfeeding. However, considerable efforts are needed to encourage and promote family, healthcare, and workplace support if Saudi mothers and their infants are to fully take advantage of the benefits breastfeeding offers.

### Limitations

Our study had some limitations. First, our participants resided in a limited geographical area, namely Riyadh, the capital of Saudi Arabia. Future research should include participants from other areas of Saudi Arabia, including rural areas, whose inhabitants may have different perspectives. Second, as this was an exploratory study to investigate factors affecting breastfeeding knowledge and awareness among Saudi mothers in Riyadh, we did not collect detailed data regarding rates of exclusive breastfeeding versus mixed feeding, or information about the initiation of breastfeeding and the introduction of formula. However, such information would be valuable and could form the basis of future research. Third, we did not specifically assess the socioeconomic status of the participants or whether they employed a nanny. While this information would undoubtedly be useful, our focus was on Saudi women of reproductive age, who may or may not have social support. This is particularly relevant for women who have relocated to Riyadh to seek better opportunities for themselves or their husbands. Again, future studies could investigate these dimensions to obtain a more comprehensive understanding. Finally, there are the researchers’ position as an “insider” in Saudi Arabian society. This could have led participants to feel mistrust toward the researchers and their motivations for conducting the study (51). Furthermore, because of the researchers’ and the participants’ shared backgrounds, potential assumptions regarding the similarity of some of their shared experiences could have led to researcher bias (52). Overall, however, we viewed having insider status to be an advantage, enabling access to the community, a good response rate, and the development of trust-based relationships between the researchers and the participants.

## Conclusion

To improve breastfeeding practices and thus improve maternal and infant health outcomes in Saudi Arabia, it is necessary to address breastfeeding facilitators and barriers. Future research should explore interventions tailored to address these factors, including investigating the effectiveness of various strategies for sustainable breastfeeding practices. We propose several recommendations to promote breastfeeding among Saudi mothers and address the barriers. Mothers’ support systems should be enhanced by promoting emotional and physical support from family members, healthcare providers, and the wider community. Improving knowledge and education through comprehensive breastfeeding education programs and information and awareness campaigns for expectant mothers and the wider community would help to dispel misconceptions and promote breastfeeding. Addressing cultural and social influences by challenging barriers to breastfeeding and promoting positive cultural beliefs that support breastfeeding is also important. Workplace and university support for breastfeeding mothers is also essential. Breastfeeding-friendly policies and facilities in the workplace and in universities can enable successful breastfeeding practices. Access to professional support, such as skilled lactation consultants and breastfeeding support services, should be available to all mothers. Furthermore, hospital policies should adhere to UNICEF and WHO’s Baby-Friendly Hospital Initiative to provide a supportive environment for breastfeeding (53), while increasing the number of certified lactation consultants through appropriate training can enhance support for breastfeeding in the community and healthcare institutions.

By implementing these recommendations, Saudi Arabia can create a supportive and enabling environment for breastfeeding mothers that will contribute to increasing breastfeeding rates and improving mothers’ and infants’ overall health and well-being.

## Data Availability

The raw data supporting the conclusions of this article will be made available by the authors, without undue reservation.
